# Clinical Manifestations and Treatment Outcomes of Pediatric Rosacea Patients: A Retrospective Study

**DOI:** 10.3390/jcm14248783

**Published:** 2025-12-11

**Authors:** Yoon Jae Kim, Jung Min Park, Hyun Mo Lee, Dai Hyun Kim, Soo Hong Seo, Hyo Hyun Ahn

**Affiliations:** Department of Dermatology, Korea University College of Medicine, Goryeodae-ro 73, Seongbuk-gu, Seoul 02841, Republic of Korea; yoonjae96@korea.ac.kr (Y.J.K.); korea2017180025@gmail.com (J.M.P.); hmhmll@gmail.com (H.M.L.); mightycell@naver.com (D.H.K.); dmsshong@gmail.com (S.H.S.)

**Keywords:** rosacea, pediatric rosacea, ocular rosacea

## Abstract

**Background:** Rosacea is a chronic inflammatory skin disorder with limited research in pediatric populations. The study aims to characterize the clinical features and evaluate treatment outcomes in Korean pediatric patients with rosacea. **Method:** We retrospectively reviewed the medical records of 22 pediatric patients with rosacea who visited a tertiary hospital in Korea (2013–2023). **Results:** A total of 22 patients (F:M = 1.75:1) were included. The mean age at presentation was 14 ± 3.3 years. Papulopustular rosacea (PPR) was the most common subtype (72.7%), followed by ocular (45.5%) and erythrotelangiectatic rosacea (ETR) (27.3%). PPR was more frequently associated with nose involvement, while ETR predominantly affected the cheek. The mean duration of systemic treatment was 16.8 weeks, with 63.6% achieving favorable responses; however, 54.5% experienced recurrence, particularly females and those with PPR. The average symptom-free duration after discontinuing systemic treatment was 16.8 months. **Conclusions:** In Korean pediatric patients with rosacea, clinical features and treatment outcomes were similar to the results of previous studies conducted in Western populations. However, in terms of epidemiology, a female predominance and adolescent onset were notable, which differ from previous studies. Additionally, the study presented the clinical differences between ETR and PPR and suggested potential predictors of recurrence in pediatric rosacea.

## 1. Introduction

Rosacea is a chronic inflammatory skin condition characterized by facial redness, telangiectasia, papules, pustules, and tissue hypertrophy. Although research on the epidemiology of rosacea is limited, rosacea prevalence has been reported between 2% and 22% [1]. Rosacea is about two to three times more common in women than men and typically affects individuals with fair skin, usually beginning in the fourth decade of life [2,3]. Rarely, rosacea symptoms can present in pediatric patients; however, the diagnosis and treatment are challenging due to the absence of established diagnostic criteria and guidelines for the pediatric population. Some epidemiological studies on pediatric rosacea report a prevalence of less than 1% [4,5,6], with one study noting an incidence of 0.89 per 1000 person-years [7]. Regarding age of onset, few studies suggest that pediatric rosacea may begin around ages four to five without gender predilection [8,9,10].

Rosacea is diagnosed clinically using two classification systems: the National Rosacea Society Expert Committee (NRSEC) system introduced in 2002 [11], and the Rosacea Consensus (ROSCO) panel system introduced in 2017 [12,13]. The NRSEC system categorizes rosacea into four subtypes based on the predominant symptoms: Erythrotelangiectatic Rosacea (ETR), Papulopustular Rosacea (PPR), Phymatous rosacea, and Ocular rosacea. While straightforward and easy to use in clinical practice, this system has the limitation of significant overlap between subtypes. Therefore, the ROSCO panel recommended a phenotype-based approach, which categorizes rosacea symptoms into diagnostic, major, or minor features, with severity assessed on a five-point scale for each phenotype. In the pediatric population, diagnostic criteria were first proposed by Chamaillard et al. in 2008, based on a small retrospective study of 20 patients [8]. However, these criteria have not been widely adopted, and classification systems established for adults are still predominantly used in clinical practice.

Similarly, treatment generally follows adult guidelines, modified according to the patient’s age due to concerns regarding side effects and safety issues [10,14]. For example, in Korea, tetracyclines are not prescribed to children under the age of 12, as they may cause permanent tooth discoloration in pediatric patients. Isotretinoin is primarily used for severe or phymatous rosacea in adults, due to concerns about premature epiphyseal closure. Treatment responses in pediatric rosacea have been reported to be comparable to those observed in adults [15].

In summary, there are limited studies on pediatric rosacea due to its low prevalence. Diagnosis and treatment have largely been extrapolated from adult guidelines. This study retrospectively analyzed Korean pediatric patients with rosacea to describe their clinical features and evaluate treatment approaches.

## 2. Materials and Methods

### 2.1. Patients

**Figure 1:**
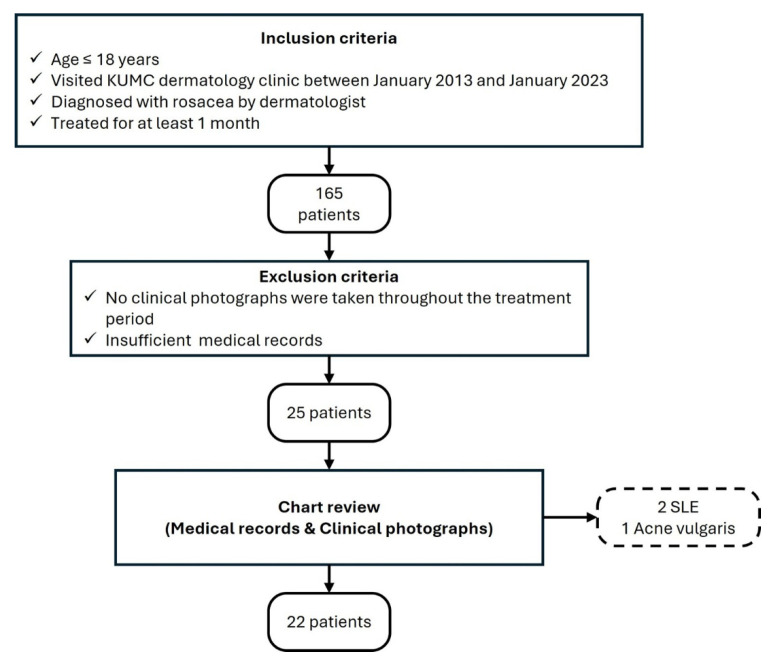
Flowchart summarizing the patient selection process for the study.

This study retrospectively reviewed patients aged ≤ 18 years, diagnosed with rosacea and treated for at least 1 month by a board-certified dermatologist at Korea University Medical Center between January 2013 and January 2023 (Figure 1). Patients without clinical photographs or sufficient medical records were excluded. A total of 25 patients initially met the inclusion and exclusion criteria. The investigators evaluated diagnosis and disease severity through the patients’ clinical photographs and collected information from electronic medical records, including sex, age, Fitzpatrick skin type (FST), duration of the condition, clinical features, and details about treatments. Upon thorough review of medical documentation, three patients were further excluded due to diagnostic misclassification. Two patients were suspected of systemic lupus erythematosus based on laboratory evaluation and were subsequently diagnosed following rheumatology consultation. Another patient was excluded because multiple comedones were identified on clinical photograph, leading to a final diagnosis of acne rather than rosacea.

### 2.2. Diagnosis and Classification System

Both the NRSEC and ROSCO panel classification systems were utilized in this study. Following the ROSCO panel recommendations, the severities of each phenotype were assessed using a five-point scale. According to the NRSEC classification system, patients were classified by their predominant clinical features, with more than one subtype assigned when multiple features were present. ETR and PPR were not classified simultaneously, whereas phymatous and ocular rosacea were allowed to coexist with other subtypes.

### 2.3. Global Severity Assessment

Overall rosacea severity was assessed before and after treatment. For patients with clinical photographs, both the Investigator’s Global Assessment (IGA) and the Rosacea Area and Severity Index (RASI) were used [16]. For those without clinical photographs at a given time point, the IGA was determined through a review of medical records.

### 2.4. Treatment

Pediatric patients with rosacea were primarily treated following adult treatment guidelines, with age-appropriated adjustments to address safety concerns and potential side effects. Tetracycline-based antibiotics are not approved for patients under 12 years old in Korea, and isotretinoin was avoided during the growth period due to concerns regarding potential effects on height and bone mineral density. Systemic agents were continued until symptom improvement was achieved, and patients were instructed to maintain topical treatments even after improvement to prevent relapse. Once symptom improvement was achieved, the dosage of systemic agents was gradually reduced and then discontinued in consultation with the patient. The treatment goal was to use systemic agents for a maximum of three to four months to achieve an IGA score of 1 or 2. Cases allowing discontinuation of therapy within this period were considered to have a favorable treatment response. For topical treatment, clindamycin, metronidazole, and ivermectin were used. Although these agents have age restrictions, they are generally considered safe in pediatric patients. In our study, clindamycin gel was recommended for papules and pustules, while metronidazole gel or ivermectin cream was prescribed for both facial erythema and papulopustular lesions.

### 2.5. Statistics

A Firth’s penalized logistic regression model was used to analyze the association between recurrence and patient characteristics. The clinical characteristics and treatment outcomes of ETR and PPR were compared using Fisher’s exact test and Mann–Whitney U test. Statistical analyses were performed with IBM SPSS version 23.0, for Windows (IBM Corp, Armonk, NY, USA), and *p* < 0.05 was considered statistically significant.

## 3. Results

A total of 22 patients (F:M = 1.75:1) were included in this study (Table 1). The mean age at presentation was 14 ± 3.3 years (median age 14). When classified by pediatric age stages, there was one patient (4.5%) in the preschool-age group (3–6 years), six patients (27.3%) in the school-age group (6–12 years), and the majority, 15 patients (68.2%) in the adolescent group (12–18 years). All patients were of Korean ethnicity. The FST distribution reflected typical characteristics of the Korean population, with no patients classified as FST I, II, or VI. The duration from symptom onset to diagnosis varied among patients, with an average duration of 14.7 ± 21.6 months (median 3 months).

**Figure 2:**
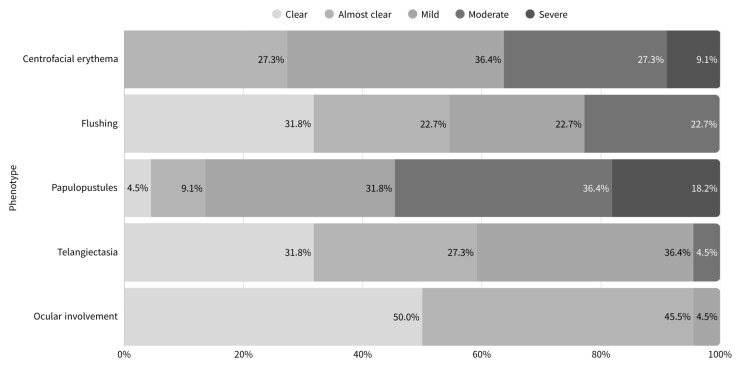
Stacked bar chart illustrating the severity of each phenotype. Patients were evaluated and classified according to the ROSCO panel recommendations. The severity of centrofacial erythema and flushing demonstrated a relatively even distribution ranging from clear to severe. The papules/pustules category showed a higher number of patients rated as moderate to severe compared to other phenotypes. On the other hand, ocular signs and telangiectasia were evaluated as presenting mild symptoms in most cases.

Table 2 summarizes the initial clinical features of pediatric patients with rosacea included in the study. Based on predominant features, the patients were classified into four subtypes. The PPR type was the most common, observed in 16 patients (72.7%), followed by the ocular type in 10 patients (45.5%) and the ETR type in six patients (27.3%). No cases of phymatous type were observed. When stratified by pediatric age groups, the single patient in the pre-school age group presented with PPR accompanied by ocular involvement. Among school-age group, one patient had ETR and five had PPR, while ocular involvement was observed in four patients. In the adolescent group, five patients were classified as ETR and 10 patients as PPR, with ocular involvement present in five cases. Additionally, patients were evaluated using the phenotype-based classification system according to the ROSCO panel recommendations. The frequency distribution of severity for each phenotype is illustrated in Figure 2, showing that the papules/pustules category exhibited a higher frequency of moderate to severe cases compared to other phenotypes. Conversely, ocular signs and telangiectasia were evaluated as presenting mild symptoms in most cases. Regarding the distribution of skin lesions, the cheek (68.2%) and nose (63.6%) were the most frequently affected areas. Ocular symptoms were observed in 10 out of the 22 patients (45.5%). Most showed mild blepharitis and lid margin telangiectasia, which improved with systemic treatment for rosacea. However, one patient required ophthalmologic care due to blepharoconjunctivitis and recurrent styes. The patient recovered without serious ophthalmic sequelae following appropriate treatment. Among the 22 patients, 20 had clinical photographs taken at their first visit, allowing for the evaluation of both IGA and RASI. The remaining two patients who lacked initial photographs were assessed for IGA based on medical chart review. The most common IGA score was 2 (mild), with 12 patients (54.5%) in this category, while five patients (22.7%) each had IGA scores of 3 (moderate) and 4 (severe). The average RASI was 13.6 ± 7.7.

Patients received a combination treatment of systemic and topical therapies. The average duration of systemic treatment was 16.8 ± 22.8 weeks (median 6.5 weeks). Eight patients required systemic therapy for more than four months (16 weeks), while 14 patients improved within four months, indicating a favorable response. Among the 22 patients, four were under 12 years old and were treated with roxithromycin. The median treatment duration was 13 weeks (range: 5–16 weeks), with all achieving an IGA score of 1. One of these patients had clinical photographs taken after treatment, allowing for RASI assessment, which showed a 69% improvement. For patients aged 12 and older, minocycline was used in four patients, while doxycycline was administered to 11 patients. The minocycline group had a median treatment duration of 7.5 weeks (range: 4–12 weeks) with all achieving an IGA score of 1, and one patient showing a 62.1% RASI improvement. In the doxycycline group, the median treatment duration was 17 weeks (range: 5–80 weeks), with three patients achieving an IGA score of 2, and the remaining achieving an IGA score of 1. One patient’s post-treatment RASI showed a 79.9% reduction. Two patients aged 17 and 18 were treated with isotretinoin for 20 and 39 weeks, respectively, both achieving an IGA score of 1. Aside from mild skin dryness, no significant side effects were reported. One patient declined systemic therapy and was treated with topical clindamycin and pulsed dye laser; however, there was no significant improvement in the IGA score. Three patients received pulsed dye laser as an adjunctive therapy in addition to systemic and topical treatments. These patients showed temporary improvement in erythema and telangiectasia but required systemic agents to adequately control rosacea symptoms. Topical treatments were prescribed according to symptoms, with 17 patients (77.3%) receiving topical clindamycin, five (22.7%) receiving topical ivermectin, and 12 (54.5%) receiving topical metronidazole. All treatments were well tolerated, with only mild irritation reported.

After treatment discontinuation, 12 patients (54.5%) returned to the dermatology clinic due to recurrence of rosacea. The time until recurrence ranged from 1 to 72 months, with an average of 16.75 ± 22.84 months (median 6.5 months). Table 3 summarizes the results of the multivariable logistic regression model analyzing the association between recurrence and patient characteristics. Due to the small sample size of 22 patients, the 95% confidence interval was quite wide. However, a statistically significantly higher recurrence rate was observed in females (OR 73.24) and in patients with PPR (OR 57.89).

Table 4 provides a summary of the clinical features and treatment outcomes of patients classified as ETR and PPR. Both ETR and PPR showed a female predominance, with no significant difference in the female-to-male ratio between the two groups. Regarding the distribution of skin lesions, PPR was significantly more likely to involve the nose than ETR (*p* = 0.011), while ETR tended to involve the cheeks more frequently (*p* = 0.149). PPR exhibited trends toward higher recurrence rates (*p* = 0.056) and greater initial disease severity (*p* = 0.115) compared to ETR. Interestingly, PPR also showed a shorter duration of systemic therapy (*p* = 0.059) and longer symptom-free periods (*p* = 0.189) after treatment. However, none of these differences reached statistical significance.

## 4. Discussion

Rosacea is a chronic inflammatory skin disease that predominantly affects fair-skinned adults between their 30s and 50s. Consequently, most clinical studies have focused on adult populations with FST I or II. The primary objective of this study was to describe the clinical features of pediatric rosacea in the Korean population and compare these findings with those reported in the previous literature. In addition, we aimed to evaluate treatment approaches and outcomes in pediatric rosacea, identifying differences from treatment strategies typically utilized in adult rosacea patients.

A recent meta-analysis on rosacea epidemiology reported a prevalence of 5.46% in adults [2]. To date, research on the epidemiology of pediatric rosacea is limited, and no studies have been conducted on the general pediatric population. However, 1–2% of all patients with rosacea are reported to be under 18 years of age [4,5]. In terms of age of onset, a single-center study involving 20 pediatric patients with rosacea aged 1–15 years reported that symptoms typically appeared at a mean age of four to five years [8]. Regarding gender distribution, meta-analysis results indicated that females accounted for 61.3% of adult cases [2], whereas in pediatric patients, occurrence was reported to be similar between boys and girls [8,9,10,14]. In this study, we focused solely on pediatric patients with rosacea, so prevalence was not determined. Among the 22 patients, 14 (63.6%) were girls, showing a gender distribution similar to that observed in adult populations. The average age at presentation was 14 years, indicating that rosacea most frequently occurred during adolescence. Differences from previous studies may be due to variations in study populations. Prior studies on pediatric patients primarily included Caucasians under 13 to 15 years, while this study included Korean patients under 19 years. We included a broader age range, with a notable number of patients aged 16 to 18 years, who were not included in previous studies. The later onset of symptoms compared to other studies could potentially be attributed to the darker skin tones in Korean patients, which may delay the visible appearance of centrofacial erythema [17]. Additionally, although female predilection can be attributed to ethnic differences, it could also be due to the higher likelihood of females visiting dermatology clinics, which may have introduced selection bias.

The prevalence of rosacea subtypes has been reported variably due to ambiguity in case definitions. In adult populations, several epidemiological studies suggest that ETR is the most common type, followed by PPR. The phymatous type, primarily observed in males, is relatively rare, while ocular rosacea occurs in approximately 25–33% of cases [3,18,19,20,21]. In contrast, the PPR type is the most common in pediatric patients, followed by ocular rosacea and ETR, with no cases of phymatous type observed. Notably, ocular rosacea can precede skin symptoms, and some studies report that up to 70% of pediatric patients exhibit ocular symptoms [8,10,14]. In this study, clinical manifestations were largely consistent with those reported in previous studies on pediatric rosacea. PPR was the most common subtype (72.7%), followed by the ocular type (45.5%) and ETR (27.3%). Unfortunately, due to the retrospective nature of the study, the temporal relationship between ocular and cutaneous manifestations could not be established. When evaluating severity based on phenotypes, the papules/pustules category exhibited higher severity scores compared to other phenotypes. Similar to adults, rosacea symptoms in pediatric patients were symmetrically distributed in the central convex areas of the face, particularly on the cheeks (68.2%) and nose (63.6%).

The pathophysiology of rosacea involves dysregulation of the innate and adaptive immune systems triggered by various environmental and endogenous factors. Upregulation of innate immune pathways (particularly the TLR2-KLK5-LL37 cascade) leads to inflammatory manifestations such as erythema and papulopustules. Persistent chronic inflammation further induces proteases, matrix metalloproteinases, and several growth factors, promoting tissue remodeling that ultimately results in telangiectasia and phymatous changes [1,22]. Based on our observations, we suggest that the limited duration of inflammation in pediatric patients may contribute to the lower frequency of secondary remodeling changes. This could partly explain why erythema and papulopustules were predominant clinical features in pediatric patients.

We used diagnostic and classification systems proposed by the NRSEC and ROSCO panel instead of the pediatric rosacea criteria proposed by Chamaillard et al. [8]. These systems facilitate clinical evaluation by classifying patients based on their phenotypes and grading the severity of each phenotype. However, an important limitation is that these criteria were originally developed for adults and may not fully reflect the unique clinical features of pediatric rosacea. First, PPR is the most common subtype in children, and this phenotype often requires differentiation from acne vulgaris, which is highly prevalent in pediatric populations. In our study, one patient initially diagnosed with rosacea was excluded after review of clinical photographs, as the presence of multiple comedones led to a final diagnosis of acne. Second, ocular involvement occurs frequently in pediatric patients and may require ophthalmologic evaluation, whereas ocular symptoms tend to be underemphasized in adult-derived classification systems. Thus, when applying adult-derived diagnostic and classification systems to pediatric rosacea, clinicians should carefully distinguish rosacea from acne and thoroughly assess ocular involvement.

Pediatric patients with rosacea were primarily treated following adult treatment guidelines, with age-appropriate adjustments. In this study, roxithromycin was used for patients under 12 years, while tetracycline-based antibiotics were prescribed for those over 12 years. Isotretinoin was reserved for moderate to severe patients who had completed their growth. Topical treatments included clindamycin, ivermectin, and metronidazole, all of which were well tolerated without significant side effects. Like systemic therapies, topical agents also have age restrictions, but they tend to be selected based on symptoms rather than age. A pulsed dye laser was attempted on four patients, resulting in temporary improvement in erythema and telangiectasia. Given that the patient who refused systemic treatment and was treated with pulsed dye laser did not show significant improvement, the laser may serve as an adjunctive treatment option. Among the 22 patients, 18 achieved an IGA score of 1, and four achieved an IGA score of 2, with 14 showing sufficient improvement in less than 16 weeks of systemic therapy. Due to the small sample size and the absence of an adult control group, statistical analysis to assess differences between pediatric and adult patients, as well as comparisons of treatment efficacy among medications, could not be performed. Nevertheless, the results of this study suggest that pediatric patients with rosacea can achieve favorable treatment outcomes comparable to those expected in adults. Notably, most patients with ocular rosacea experienced improvement in ocular symptoms with standard rosacea treatment and did not require additional ophthalmologic interventions. Previous studies also indicate that tetracycline-based antibiotics and macrolides are effective in managing pediatric ocular rosacea [23,24]. However, in pediatric patients, particularly females, the risk of corneal involvement remains relatively high [15]. Therefore, prolonged antibiotic treatment may be necessary, and regular ophthalmologic assessments are recommended for pediatric patients with rosacea. Based on our clinical experience, ophthalmologic consultation is warranted when patients develop discomfort due to styes or when inflammation extends beyond the eyelid margin to involve ocular structures such as the conjunctiva, cornea, or sclera.

More than half of the patients returned to the dermatology clinic due to symptom recurrence. Given that patients with mild symptoms are less likely to revisit a tertiary hospital, the actual recurrence rate is probably higher than reported. The multivariable logistic regression model identified statistically significantly higher recurrence rates in females and the PPR subtype, but the confidence intervals were very wide. Further research will be needed to confirm these findings.

The clinical differences between ETR and PPR have been a topic of significant interest, though they are not fully understood. In 2013, Tan et al. published a study on clinical associations between rosacea subtypes in adults aged 18 years and older [25]. According to their findings, PPR was more frequently associated with burning/stinging sensations, phymatous changes, and edema compared to ETR. In contrast, skin dryness was more commonly observed in ETR [25]. In this study, we compared the clinical characteristics and treatment outcomes of pediatric patients with ETR and PPR. A significant association was found between PPR and nose involvement compared to ETR, while cheek involvement was more frequently observed in ETR, though this was not statistically significant. Notably, despite patients with PPR presenting higher initial disease severity and recurrence rates, they required a shorter duration of systemic therapy compared to ETR. Although these results did not reach statistical significance, they suggest that in pediatric rosacea, papulopustules may respond more rapidly to systemic treatments but are more prone to recur. These results indicate that sustained remission and recurrence prevention are particularly important in pediatric rosacea patients with PPR subtype. Gradual tapering of systemic medications, continued use of topical therapies, and strict lifestyle modification should be considered as part of long-term management.

In conclusion, this study identified several clinical characteristics of pediatric rosacea in the Korean population that differ from those reported in previous studies. Differences between the ETR and PPR subtypes, as well as potential predictors of recurrence, were also demonstrated. To the best of our knowledge, there have been no published studies specifically addressing pediatric rosacea patients with skin of color. This study is significant as it provides an analysis of the clinical features and treatment outcomes of rosacea within a Korean pediatric patient. However, the study has inherent limitations related to its small sample size and retrospective design. Therefore, caution is required when generalizing the results to the broader pediatric population. Future large-scale, well-designed studies or meta-analyses are warranted to validate these results.

## Figures and Tables

**Table 1:** Demographic data of the patients included in the study.

Sex (F:M)	1.75
Male	8 (36.4%)
Female	14 (63.6%)
Age (Years)	14 ± 3.3
	Median (min–max): 14 (6–18)
Preschool (3–6 years)	1 (4.5%)
School (6–12 years)	6 (27.3%)
Adolescent (12–18 years)	15 (68.2%)
Fitzpatrick skin type	
I	0 (0.0%)
II	0 (0.0%)
III	9 (40.9%)
IV	8 (36.4%)
V	5 (22.7%)
VI	0 (0.0%)
Duration (months)	14.7 ± 21.6 Median (min–max): 3 (1–72)

**Table 2:** Clinical features of pediatric patients with rosacea included in this study.

Subtype	Number of Patients
ETR	6 (27.3%)
PPR	16 (72.7%)
Phymatous	0 (0.0%)
Ocular	10 (45.5%)
Location	
Cheek	15 (68.2%)
Nose	14 (63.6%)
Perioral	2 (9.1%)
Forehead	1 (4.5%)
Full-face	4 (18.2%)
Ocular signs	10 (45.5%)
Lid margin telangiectasia	4 (18.2%)
Blepharitis	5 (22.8%)
Blepharoconjunctivitis	1 (4.5%)
Minor features	
Burning/Stinging	8 (36.4%)
Edema	9 (40.9%)
Dryness	6 (27.3%)
Severity index	
IGA	2.7 ± 0.8
0 Clear	0 (0.0%)
1 Minimal	0 (0.0%)
2 Mild	12 (54.5%)
3 Moderate	5 (22.7%)
4 Severe	5 (22.7%)
RASI	13.6 ± 7.7

ETR Erythrotelangiectatic rosacea, PPR Papulopustular rosacea, IGA Investigators Global Assessment, RASI Rosacea Area and Severity Index.

**Table 3:** Multivariable logistic regression model assessing associations of patient characteristics with recurrence. A significantly higher recurrence rate was observed in females, with an odds ratio (OR) of 73.24, and in the PPR, with an OR of 57.89.

	*p*-Value	Odds Ratio	95% Confidence Interval	
			Lower Limit	Upper Limit
Sex				
F	0.036	73.24	1.33	>999
M	NA	1	NA	NA
Subtype				
PPR	0.037	57.89	1.28	>999
ETR	NA	1	NA	NA
Age	0.138	1.45	0.89	2.42

F Female, M Male, PPR Papulopustular rosacea, ETR Erythrotelangiectatic rosacea.

**Table 4:** The clinical features and treatment outcomes of ETR and PPR were compared. For continuous variables, data were presented as the median (interquartile range). The statistical analysis revealed that PPR was significantly more likely to involve the nose. Although there was no statistical significance, PPR exhibited higher recurrence rates, greater initial disease severity, shorter duration of systemic therapy, and longer symptom-free periods.

	ETR (n = 6)	PPR (n = 16)	*p*-Value
Sex (F:M)	2	1.67	1.000
Location (%)			
Cheek	83.3	37.5	0.149
Nose	16.7	81.2	0.011
Forehead	33.3	25	1.000
Perioral	16.7	31.3	0.634
Major features (%)			
Flushing	66.7	68.8	1.00
Ocular sign	33.3	56.2	0.635
Minor features (%)			
Burning/Stinging	33.3	36.5	1.000
Edema	33.3	43.8	1.000
Dryness	16.7	31.3	0.634
Recurrence (%)	16.7	68.8	0.056
Age (years)	13.50 (12.25–18.00)	14.00 (12.00–17.00)	0.802
Global severity			
IGA	2.00 (2.00–2.25)	3.00 (2.00–4.00)	0.115
RASI	10.05 (8.93–12.22)	14.15 (7.35–20.65)	0.444
Duration of systemic treatment (months)	17.50 (15.50–30.75)	9.50 (5.00–18.50)	0.059
Symptom-free period (months)	1	7.00 (3.00–34.00)	0.189

F Female, M Male, ETR Erythrotelangiectatic rosacea, PPR Papulopustular rosacea, IGA Investigators Global Assessment, RASI Rosacea Area and Severity Index.

## Data Availability

The data supporting the findings of this study are available from the corresponding author upon request.
